# An Introduction to the Combustion of Carbon Materials

**DOI:** 10.1002/chem.202200117

**Published:** 2022-07-25

**Authors:** Emmanuel Picheau, Sara Amar, Alain Derré, Alain Pénicaud, Ferdinand Hof

**Affiliations:** ^1^ Centre de Recherche Paul Pascal CRPP UMR5031-CNRS/ Université de Bordeaux 115 Avenue du Dr Albert Schweitzer 33600 Pessac France

**Keywords:** carbon nanomaterials, combustion, diffusion, kinetics, surface reactions

## Abstract

Combustion is arguably as old as homo sapiens ability to observe and use fire. Despite the long tradition of using carbon combustion for energy production, this reaction is still not fully understood. This can be related to several facts that are intertwined and complicate the investigation, such as the large variety of possible carbon structures, the actual surface structure, porosity, the solid‐gas nature of this reaction, diffusion limitation and fundamental reaction steps. In this review, a brief history of carbon combustion science is given, followed by a detailed discussion of the most important aspects of carbon combustion. Special attention is given to limitations for example diffusion. In carbon combustion, kinetic control can rarely be observed. The literature of the fundamental reaction steps actually occurring on the carbon framework is reviewed and it becomes apparent that the reaction is occurring primarily on defects on the basal plane. Thus, the reaction between oxygen and carbon may be used as an analytical tool to provide further insights into novel materials, for example synthetic carbon materials, fibres and graphene type materials. Mastering the combustion reaction in all its complexity may prove to be very valuable in the future.

## Graphical abstract

**Looking back over centuries** of research on carbon combustion, this reaction has literally fuelled the development of mankind and truly astonishing technological advancement. Carbon combustion is complex and interplay of different limitations such as diffusion, heat/mass transfer, and reactivity of active sites is observed. The understanding and a unifying concept of carbon combustion may prove to be valuable also for the understanding of novel carbon nanomaterials, such as fibres or graphene like materials.

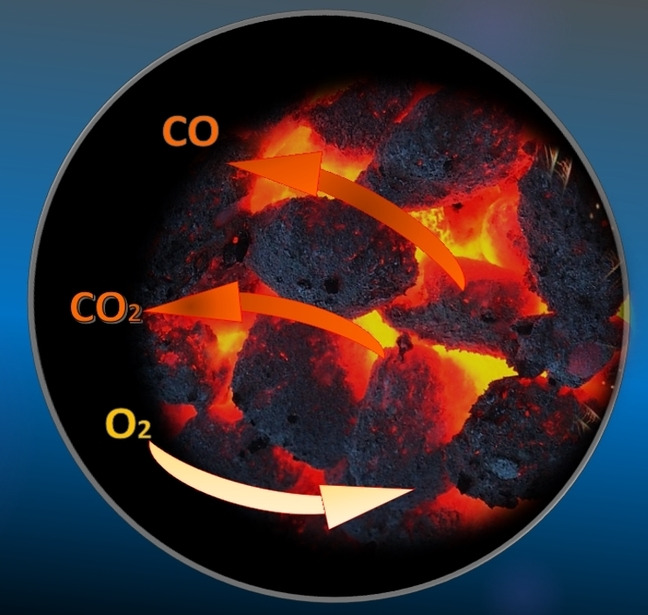

## 1 Introduction

### 1.1 Starting with fire

Combustion is arguably as old as homo sapiens ability to observe and use fire. Combustion started at least about 4*10^5^ years ago with burning wood and later coal as a means to provide heat in cold times and regions and allowed humankind to spread over the planet.[^1^, ^2^] Nowadays, combustion is defined as an exothermic oxidation of a fuel by an oxidant. The technical exploitation of combustion reactions has fueled various technical breakthroughs in the history of humankind, for example advanced metallurgy^[3]^ and the development of the steam engine.^[3]^ These are important milestones that have enabled the lifestyle we know today. Carbon was defined and named as an element by Lavoisier in 1789 to replace coal, and a distinction between the naturally occurring fossil fuel and the element, which it is made of, was hence established.^[4]^ According to H. Marsh, carbon materials have always been the friendliest of materials, accounting in early days for writing, heat and light as well as some medical uses (the usage as key ingredient for gunpowder may not been evaded though).^[5]^

At the end of the XVIII^th^ century, the works of Priestley, Scheele and Lavoisier lead the latter to conceptualize combustion as the reaction of an inflammable substance with air, or a part from air, that he called oxygen.^[4]^ Nowadays, combustion is a high tech technology found in various applications ranging from heat shields of rockets to modern coal plants. Despite modern days push for cleaner technologies in terms of CO_2_ emission, roughly a quarter of the world electricity is still produced in industrial plants such as pulverized fuel plants^[6]^ and coal combustion remains first in the electricity mix (35.1 % of the worldwide electricity production in 2020).[^7^, ^8^]

As simple as it may appear, carbon combustion or the reaction of carbon and oxygen to yield carbon monoxide and dioxide, is known for its complexity.^[9]^ The following is a brief tentative to review the state of the art of carbon combustion and the challenges it still encompasses.

### 1.2 Classification of carbon materials

**Figure 1:**
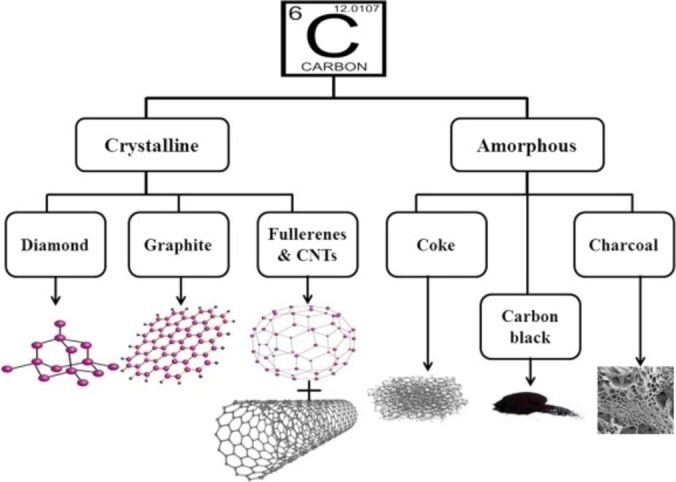
Classification of some carbonaceous materials from reference.^[10]^ Depicted are crystalline forms of carbon such as diamond graphite and nanoforms such as fullerene and carbon nanotubes (CNTs). Moreover, there are also various carbon forms that exhibit an amorphous structure, such as cokes, carbon black and charcoal. Adapted with permission from Ref. [10]. Copyright 2022, Wiley‐VCH.

Carbon is the building block of life due to the large variety of binding motifs possible. This is caused by its four valence electrons, that are capable of mixing atomic orbitals (AO, 2 s and 2p) under formation of hybrid orbitals (HO): *sp*, *sp* ^2^ and *sp* ^3^. The overlapping of HO of carbon atoms leads to the formation of strong sigma bounds, while (if present) the remaining p AO form π bonds. To properly apprehend the complexity of combustion reaction of carbon materials, it is first necessary to understand how diverse this class truly is. Carbon is one of the few elements occurring in its elemental form on Earth, in contrast to the majority of other elements which can be typically found in molecular form for example as oxides. Amorphous forms such as black coal and anthracite and the two macroscopic crystalline forms, graphite and diamond are known to most human beings nowadays. The expression “carbon materials” contains much more different constituents forming each a different sub‐class of materials (Figure 1).^[10]^

#### 1.2.1 Crystalline forms

In case only carbon atoms are present, an assembly of *sp* ^3^ hybridized carbons (tetrahedral geometry) will form a face centred cubic crystal, the diamond. If only *sp* hybridized carbon atoms are involved, it may form the hypothetical carbyne.[^11^, ^12^] If *sp* ^*2*^ hybridized carbon atoms are solely present, a planar hexagonal motif, the graphene layer, is generated. A stacking of graphene layers causes interaction of π orbitals (Van der Waals interaction) and results in the well‐known graphite. Depending on the stacking order, relationship between the individual layers in respect to each other, different forms of graphite can be distinguished for example Bernal (AB stacking) or turbostratic (random stacking). Highly oriented pyrolytic graphite (HOPG), a synthetic material, exhibiting high structural ordering along the c‐axis is obtained by compressing a carbon precursor at high temperature in vacuum.^[13]^ It is one of the standard materials used for investigating the fundamental reaction steps, due to the reproducibility of its structural parameters. Therefore, some important studies on carbon combustion and the evolution of the carbon structure are based on HOPG.[^14^, ^15^, ^16^, ^17^, ^18^, ^19^, ^20^, ^21^, ^22^, ^23^, ^24^]

Amazingly late in the history of science, at the end of last century, two novel allotropic forms of carbons were discovered, namely fullerenes^[25]^ and carbon nanotubes.^[26]^ Fullerenes correspond to the arrangement of pentagons and hexagons forming a nanometric sized spherical macromolecule.^[27]^ Carbon nanotube is a class of materials being cylinders of nanometers range diameter, made of carbon hexagons as repetition motif.

Carbon materials, by no means, are restricted only to the two classical allotropic and the crystalline nano forms since various other materials are known, which originate either from nature like coals or are synthetic such as carbon black.

#### 1.2.2 Amorphous forms

**Figure 2:**
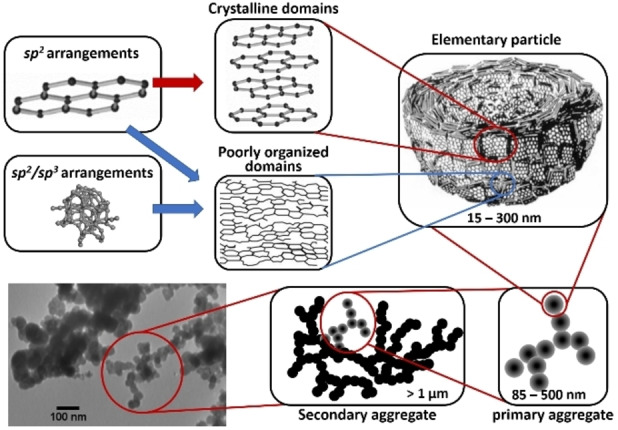
General structure of carbon black. Elementary particles are first made of randomly oriented graphitic crystallites (containing sp^2^ hybridized carbon) and of amorphous domains (containing sp^2^ and sp^3^ hybridized carbon) of varying size, domain size, crystallinity, defect density, etc. Covalently linked together, they form indivisible primary aggregates of various sizes. Less cohesive secondary aggregates are finally formed by Van der Waals interactions between primary aggregates. Adapted with permission from Refs. [28–31]. Copyright 2022, Wiley‐VCH.

Carbon blacks, a commonly used and studied class of materials, are almost purely carbon composed.^[12]^ They possess a quite complex multi‐scale structure detailed on Figure 2,[^28^, ^29^, ^30^, ^31^] and may serve as an educational example for solid carbon structures because the same understanding principle might be applied to cokes, and other synthetic disordered carbon (nano)‐materials.^[30]^ Elementary particles are first made of randomly oriented graphitic crystallites of varying size, domain size, crystallinity, defect density, etc. Covalently linked together, they form indivisible primary aggregates of various sizes. Less cohesive secondary aggregates are finally formed by Van der Waals interactions between primary aggregates. This overall structure generates a porosity, possibly on the nano, meso, and macro‐scale.

**Figure 3:**
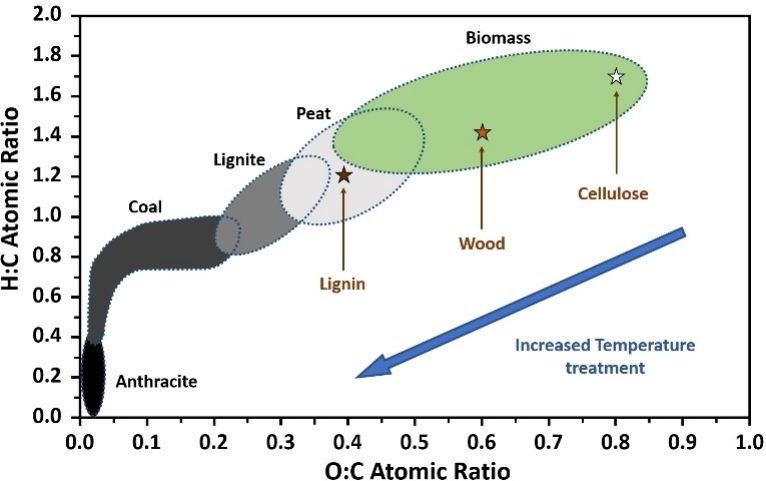
Van Krevelen diagram for various solid fuels. Data extracted from reference.^[32]^ By plotting the hydrogen:carbon atomic ratio as a function of the oxygen:carbon atomic ratio, the  origin and maturity of different carbon based materials can be assessed. It is a common technique to classify a natural material into a specific group for potential applications e.g their heating value for power plants. Energy density values increase following the same arrow that the increased temperature treatment on the diagram.

Coal originates on the other hand from the decomposition of biomass, and different classifications have been established ranging from peat, over low rank coals (e. g. lignite) over medium rank coals (e. g. brown coal) to high rank coals (e. g. anthracite), see Figure 3.^[32]^ While typically in low rank coals the oxygen content is high (>30 %) and the carbon content somewhat low, in anthracite the carbon content reaches values close to 100 %.[^33^, ^34^] Anthracite exhibits an extended *sp* ^2^ carbon content and is structurally related to graphite. The transition between anthracite and graphite occurs only under the application of compressive force.[^35^, ^36^] In contrast to crystalline materials such as graphite, the classification of coals, does not imply any type of strict ordering but is based on a common property for example heating value, carbon to oxygen ratio, or a geographical mining place. Due to the large difference in binding motifs between the different types of coals from different places in the world, even if they are in the same classification, their chemical properties may be tremendously different. In coals, the structural picture becomes even more complex due to the large content of possible heteroatoms, (H,O,N,S, etc.). By pyrolysis, carbon content and crystallinity of a given coal can be increased, consequently a new material is formed. There is, as a result, a myriad of possible amorphous carbon structures, which gets more complex through pyrolysis.[^12^, ^37^] Thus, carbon materials vary widely in structure, texture, bonding, porosity, properties and possible exploitation scenarios. As very well known in the material science field, materials reactivity is directly linked to the structure. With such variety of potential structures, a clear behaviour of carbon materials combustion becomes challenging to describe. Thus, for the combustion of specific carbon forms on an industrial scale, a detailed optimization process is required balancing competing factors, such as thermal efficiency, flow rates, etc. This picture is even more complicated if the heteroatom content, porosity, and heat transfer are considered. Moreover, there are synthetic forms such as cokes, carbon blacks and activated carbons, whose structural complexity matches those of coal and their properties are intrinsically linked to their chemical structure and porosity. In analogy to the discussed classification of coals, those carbon forms are often grouped by their respective production conditions/method for example lamp black or furnace black.^[38]^ Another type of classification is based on the type of respective parent material for example activated carbon is usually derived from charcoal, whereas activated coal is derived from coal. Many derivatives that in some shape or form can be related to fullerenes (graphitic shells, carbon spheres, onion structures, etc.), are known which contain various degrees of pyramidalization angles and/or binding forces.^[39]^ All those carbon materials, synthetic or natural, are tremendously important to various technologies today, for example energy production, metallurgy, or as filler materials, supercapacitors, and absorbers.^[40]^ In conclusion, classification of carbon materials is based either on specific properties or synthesis methods rather than structural uniformity. Thus, when comparing kinetic data of different type of carbon materials such as coals, or cokes the reader should be aware that the underlying structure, porosity and chemical properties may be vastly different from one material to the other and consequently from one study to another.

### 1.3 Structure‐property relationships and importance of defects

In carbon materials, each kind of bonding motif possesses different geometrical orientation, resulting in different reactivity to each other. To demonstrate the structure to property relationship, arguably the best examples are diamond and graphite, both pure, crystalline forms of carbon. Diamond is one of the hardest materials known, transparent and insulating, while graphite is a lubricant, shiny grey and an electrically conducting material. Their divergent properties are readily explained by their respective bonding motifs. As the total energy is the sum over all interaction of all carbon atoms,^[31]^ different stacked graphite will exhibit different reactivity versus surface reaction such as combustion. From a macroscopic, bulk point of view, a perfect graphite crystal does not exist. Graphite samples exhibit multiple crystallites in possibly different orientation (grains) linked by grain boundaries (line defect) where a distortion of *sp* ^*2*^ HO can lead to a non‐hexagonal arrangement along a line.^[41]^ Graphite and graphitic materials also contain a certain number of other defects, such as *sp* ^*3*^ hybridized carbon (point defect), dislocations (for instance edge dislocation) or diene defects (an arrangement of a pair of pentagons and a pair of heptagons), usually called Stone‐Wales defects.^[42]^ All these defects preserve the global planarity of the layer, and thus are hard to distinguish by crystallographic methods. Thus it is important to note that atoms involved in any kind of defect won't have the same reactivity than perfectly *sp* ^2^ hybridized basal plane carbon atoms.^[43]^ When it comes to carbon combustion, defects play a major role, as discussed in Chapter 6. Graphite materials may differ also in grain size, and this is a further crucial parameter for their reactivity i. e., different combustion kinetics. Difference in grain size are of course present in all types of carbon materials, for example also in nano carbon or amorphous carbon.^[12]^ The particle size is typically determined by sieving, whereas the crystallographic grain size is determined by Raman spectroscopy and X‐ray Diffraction (XRD). However, it is clear that especially for nanosized carbon materials, the application of those methods and the interpretation of the derived data become challenging. With such a variety of carbon materials, all possessing different properties as a result of different structures, it is not surprising that very different kinetic behavior has been reported for the carbon combustion reaction.^[9]^ This is even more pronounced with materials which are sorted rather by synthesis condition than structural parameters, for example pitch tars, coals and coke. With a general lack of deep structural analysis in the related literature, it is tremendously difficult to sort between materials studied and derived trends. The interest in graphene related materials, and the development of new analytical methods to study carbon surfaces, especially defects may help in this endeavor.[^44^, ^45^, ^46^, ^47^, ^48^, ^49^]

## 2 Carbon Combustion: A Lever of (R)Evolutions – The Reaction in Literature

### 2.1 Carbon combustion through history

**Figure 4:**
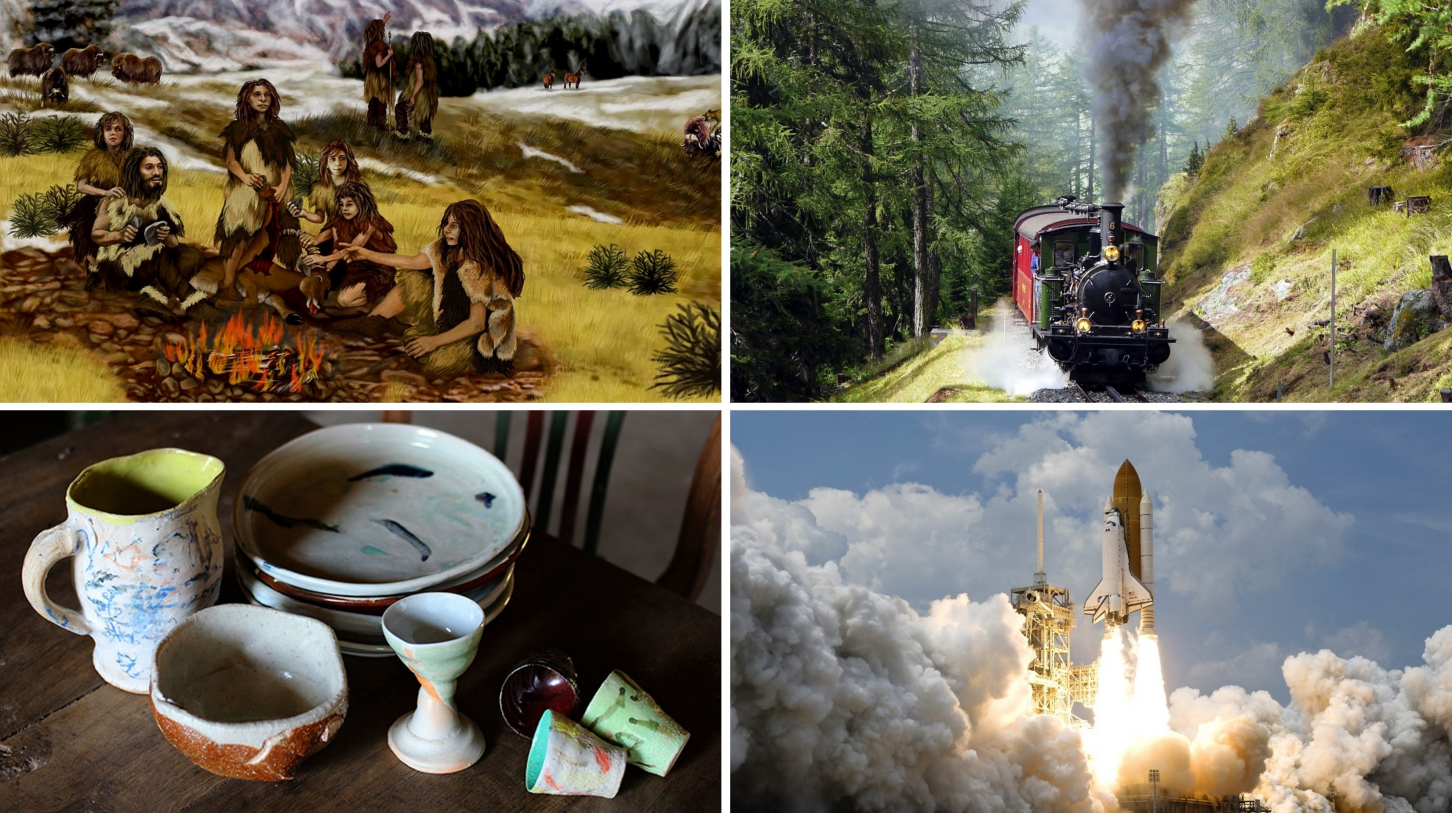
Use of combustion reactions over the centuries. Combustion reaction is used in pottery, heat generation, as well as fuel for different types of engines. It is truly one of the central technologies of mankind that is used from ancient times until today.^[55–57]^ (Credit for ceramics picture: Brigitte Pénicaud).

Although chemistry was not considered to be a scientific discipline before the end of the 18^th^ century, the use and study of carbon combustion in human history is much older and arguably started with the discovery of fire.[^50^, ^51^] Since then, carbon combustion has been the foundation for numerous technological (r)evolutions in the history of human kind.^[52]^ Of particular usefulness was the combustion of charcoal, obtained after pyrolysis of wood or originating from biomass degradation, for both the heat and reductive atmosphere that it generates. For example, conception of ovens based on charcoal combustion allowed evolution of ceramic fabrication over the millennia; the oldest known ceramic being made more than 20000 years ago.^[53]^ Having started about 6000 years ago with the reduction of ores into copper by carbon monoxide (obtained from the combustion of charcoal above 700 °C), metallurgy progressed in line with carbon combustion domestication. Directly arising from charcoal combustion in bloomery type of furnaces,^[54]^ mastery of smelting process gave rise successively to the bronze age (about 2500 BC) and the iron age (1000 BC) (Figure 4[^55^, ^56^, ^57^]). The driving force was to reach higher and higher temperatures during combustion to meet the temperature demand for the respective redox reactions. This was achieved by careful selection of the combustion material, and design of the smelters to tune the air‐flow in order to avoid convection. Later, at the beginning of the 19^th^ century, the industrial revolution was partially driven from the incremental advancements during the past centuries of the blast furnace first burning cokes, then anthracite, which allowed the production of steel.[^58^, ^59^, ^60^] Here, once again a selection of carbon materials towards higher carbon content was combined with improved reactor design/packaging. Over the centuries this endeavor has led to an improved reactor design, providing more and more oxygen to the reactor, avoiding convection as much as possible, to be able to burn carbons with higher carbon content efficiently. A second incontestable breakthrough which lead to the industrial revolution was the steam engine, pulling its driving force from carbon combustion. While for long time in history neither the question of thermodynamic efficiency nor the type of fundamental reaction steps was essential, the major quest was to reach higher and higher temperatures. The difficulty here is to maintain a constant burn off ratio, as anthracite is ultimately much harder to burn than charcoal or peat. The reasons and concepts behind this observation will be discussed in the following chapters of this review. It shall not be omitted that the optimization of carbon combustion on an industrial scale is always a matter of balancing competing factors such as intrinsic reactivity and partial pressure amongst others.

### 2.2 An industrially driven research

Over the centuries, the use of the carbon combustion reaction to improve the human way of life was essentially an industrial endeavor.^[52]^ This aspect is largely reflected in the scientific literature, especially from the last century during which the majority of studies focused on answering the specific industrial needs of a given time. Those needs tailored research and development in a certain way, for instance by optimizing a specific type of anthracite for a combustion process by reducing the grain size. From a heat generation engineering point of view, the reaction can be decomposed in three different steps: the start of the reaction, which requires a certain amount of energy (temperature) for initiation. This is followed by the propagation where the heat generated by the exothermic reaction with oxygen is sufficient to maintain a steady‐state. This step is characterized by a constant mass loss rate for the carbon material. If the temperature decreases or the amount of reactant is insufficient, the reaction stops, what correspond to the third step.^[61]^ It is certainly visible that following this approach, an optimization of power plants or steel industry, is readily feasible. A deep knowledge and understanding of the reaction, for example the resolution of the chemical fundamental reaction steps, is indeed not needed. From an engineering point of view, kinetic parameters such as activation energy are vital to maximize the efficiency for specific solid fuels and help to choose the best material for a given industrial set‐up (or design the best reactor for a given carbon type). This need is reflected by a large amount of studies on specific coals and cokes from various origin and sources.[^62^, ^63^, ^64^, ^65^, ^66^, ^67^, ^68^, ^69^, ^70^] Combustion of carbon materials was also extensively studied in the framework of nuclear power plants. Most nuclear reactors contain the so‐called nuclear graphite as a structural material to mediate the reaction. A detailed review on this important material can be found in reference.^[71]^ Under normal conditions, nuclear graphite is exposed to elevated temperature in an oxygen free environment. Many studies have then been conducted on nuclear graphite to anticipate catastrophic scenarios of unexpected air ingress, and gain predictive knowledge for detrimental worst case scenarios.[^71^, ^72^, ^73^, ^74^, ^75^, ^76^, ^77^, ^78^, ^79^] Carbon/carbon composites and pyrocarbon in aeronautic/aerospace industries are high technology products serving as thermal shield, brakes, carbon fibers, etc. Their technological feasibility is based on various studies on carbon combustion as well.[^80^, ^81^, ^82^, ^83^, ^84^, ^85^, ^86^, ^87^, ^88^, ^89^] This research is in majority application oriented, optimizing the carbonization step in such a way that a certain material property is yielded for example thermal resistance or thermal conductivity. As mentioned earlier, research on combustion of nano carbon forms (carbon nanotubes, soot, carbon nanospheres, etc.) results from their more recent discovery.[^90^, ^91^, ^92^, ^93^, ^94^, ^95^, ^96^, ^97^, ^98^, ^99^, ^100^, ^101^] Beyond the simple study of nanocarbon combustion, the reaction has been used several times in the past years as a synthesis tool for new materials and hybrids such as functionalized biomass char for sorption purposes,^[102]^ nickel coated carbon for lithium and sodium storage,^[103]^ or to do metallurgy at the nano particle scale.^[104]^ Moreover, carbon combustion has been also exploited for purification purpose^[105]^ and material modification, for example oxidative opening of carbon nanotubes^[106]^ as well as to increase porosity in carbonaceous electrode materials. Overlooking the literature of the last decades, another point emerges readily: the variety of used oxidant. It is indeed possible to find studies of carbon oxidation with molecular oxygen, eventually from wet and dry air,[^67^, ^71^, ^107^, ^108^] atomic oxygen,[^109^, ^110^, ^111^, ^112^] steam,^[113]^ and CO_2._[^66^, ^114^, ^115^, ^116^] For each oxidant, the total pressure,^[117]^ oxidant partial pressure^[118]^ and temperature range has been varied in a broad range.^[119]^ In this apparently vast experimental variation, a general coherence or continuity between studies is lacking. As a result, the drawing of a general picture on carbon combustion from industrial driven studies, by far the majority of the literature, is indeed challenging.

### 2.3 Fundamental approaches

Given its importance for human technology, it is not surprising that carbon combustion had a role in the definition of chemistry as a science. It suffices to mention Lavoisier enouncing the mass conservation principle and burying at the same time the phlogiston, a previous alchemy theory of combustion, to illustrate it.^[120]^ Despite the picture of industrially driven research encompassed in the previous paragraph, the carbon combustion reaction has also progressed significantly from a fundamental aspect since Lavoisier. One of the first fundamental aspects that caught the interest of scientists was if CO, CO_2_, or both, is produced by the primary reaction between oxygen and the carbon surface. In 1915, Irving Langmuir was writing that this “has long been a disputed question” and proposed a low‐pressure experiment to answer it definitively.^[121]^ This question continued however to be asked over decades[^119^, ^122^] and it has been observed time and time again that the ratio between primary production of CO and CO_2_ changes in dependence to several experimental parameters. Several strategies to avoid, minimize or delay secondary reactions have been employed among them, the use of low pressures, low temperatures, high flow rates and inhibitors.^[122]^ The latter, which was first proposed by Arthur, allows to ascertain that both species are primary products.^[123]^ This has been confirmed several times since then by isotopic studies (see section 6).^[124]^ Arthur also proposed that the CO/CO_2_ ratio strongly depends on the temperature and proposed an exponential law to account for it.^[125]^

**Figure 5:**
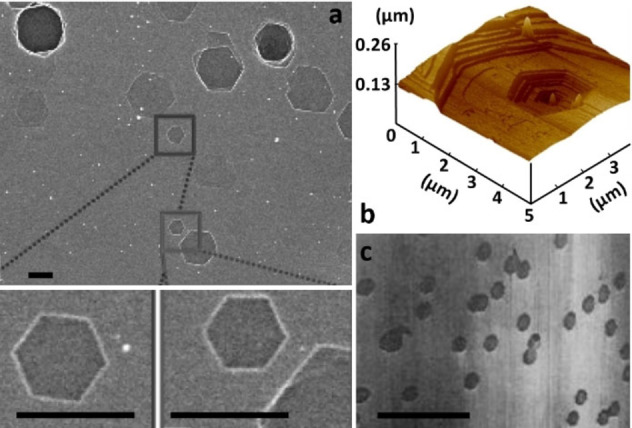
SEM (a), AFM (b) and STM (c) images of HOPG surfaces after partial oxidation. Value for the scale bar (a and c) is 1 μm. Carbon combustion occurs not randomly on the carbon basal plane but is occurring on specific sites (also called active surface sites). Typically, the reaction occurs in proximity to the initial active sites due to progressive formation of reactive sites in the course of the carbon combustion (see also chapter 6 for a more detailed discussion). Adapted with permission from Ref. [20, 23, 24]. Copyright 2022, Wiley‐VCH.

This lead to the development of several empirical models to account for the thermal evolution of the ratio between CO and CO_2_, exemplary by J. R. Arthur (cf section 6).[^123^, ^125^, ^126^, ^127^] The dependence of products distribution on other parameters such as oxygen content, presence of impurities, burn off value or carbon type has been also studied by several authors. Remarkably, attempts to understand the influence of the carbon material on the product distribution, led some authors (among them from the Walker's group) to question the link between the structure of the material and its combustion rate. Another important concern has been to link the structure of the material with its combustion rate. This is reflected by different attempts to normalize the combustion rate of any carbon material by a structural parameter, potentially allowing the definition of a master curve independent of the carbon source. In those endeavors, different techniques to measure the surface area have been developed and concepts of active and reactive surface area, or active sites have been applied.[^128^, ^129^] Arising with the development of electron microscopy and later near‐field microscopy, several authors have studied the progress of oxidation on a carbon surface by imaging sequences of pit growth on the surface over time (see Figure 5).[^14^, ^15^, ^16^, ^17^, ^18^, ^19^, ^20^, ^21^, ^22^, ^23^, ^24^] A third, major albeit more recent direction, is connected to the understanding of formation of the surface complex, a prerequisite to solve the fundamental reaction mechanism.[^124^, ^130^, ^131^, ^132^, ^133^, ^134^] Among the scientists who dedicated their research to the understanding of carbon combustion are P. L. Walker,[^85^, ^107^, ^108^, ^114^, ^115^, ^117^, ^118^, ^119^, ^128^, ^133^, ^135^, ^136^, ^137^, ^138^, ^139^, ^140^, ^141^, ^142^, ^143^, ^144^, ^145^, ^146^, ^147^, ^148^, ^149^] and his former student L. R. Radovic.[^97^, ^150^, ^151^, ^152^, ^153^, ^154^, ^155^, ^156^, ^157^, ^158^] They tackled various aspects of this complex reaction over several decades. In 1959 Walker et al. published what we believe to be one of the first exhaustive reviews on carbon combustion.^[122]^ J. M. Thomas, another scientist who has worked on the fundamental understanding of the reaction, published a referential paper in 1970.^[159]^ For a more recent overview over fundamental and technical aspects of the carbon combustion, the less novice reader might be also referred to recent publications and review articles that cover specific models in more detail.[^9^, ^71^, ^74^, ^160^]

## 3 Carbon Combustion in a Nutshell – Focus of this Review

Despite the fact that carbon combustion has been the driving force in countless technological breakthroughs, this reaction poses still a significant challenge for scientists. This is partly driven by the success of (chemical) engineers, which allowed exploiting carbon combustion in fascinating applications as discussed above. On a fundamental perspective, carbon combustion appears to be simple, but the fundamental reaction steps are nothing of the same. This can be understood in the context of three main aspects of this topic, *i)* the structural variety carbon materials exhibit, *ii)* the reaction being a solid‐gas type of reaction, *iii)* the complex, subsequent chain of reaction, starting from the adsorption of oxygen on the carbon plane, over the breaking of carbon bonds, to desorption of the gaseous products. Naturally, these 3 aspects are occurring in every sample simultaneously, which might explain the difficulties of formulating a concise theory covering all possible aspects and materials. In the following paragraphs, the basic concepts of these three aspects will be discussed briefly, without trying to be comprehensive in each detail. The interested reader might be referred to more specialists’ reviews on topics such as porosity, structural investigation, heat/mass transfer, and reactor design to reduce mass transfer limitations.[^62^, ^65^, ^161^] Details on the design and use of reactors for industrial carbon combustion can be found in references.[^162^, ^163^, ^164^] Solutions for carbon capture technologies can be found in references.[^165^, ^166^]

**Figure 6:**
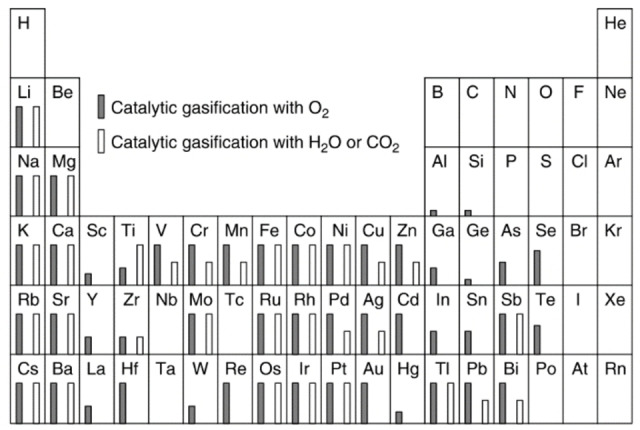
Catalytic effect of inorganic component on carbon combustion. A large variety of different elements, especially transition metals atoms, are capable of catalyzing combustion reaction with oxygen but also with water or CO_2_ which is of crucial importance when discussing kinetics or reaction mechanism. Adapted with permission from Ref. [167]. Copyright 2022, Wiley‐VCH.

The structural variety ends not here, as carbon can be readily bound to heteroatoms (H, N, S, O, etc.). In carbon materials that originate from biomass, this content has a significant impact on the combustion properties.^[167]^ It is also noteworthy that the majority of d‐block elements are able to catalyze the carbon oxidation reaction (Figure 6, adapted from ref.^[167]^),[^141^, ^167^, ^168^, ^169^, ^170^] and it is noteworthy that transition metals are frequently present in naturally occurring carbon materials.

Catalytic effects change the mechanism of carbon combustion significantly, thus we will limit our discussion in the next few paragraphs on the simplest case, the reaction of pure carbon with molecular oxygen. The interested reader is referred to review articles discussing the catalytic impact on carbon combustion.[^167^, ^168^, ^170^] The porosity, formed by loose aggregation of individual particles, is the reason why carbon combustion is often limited by diffusion effects. We will discuss the importance of the surface effects, and why mass transfer limitations are typically observed for the carbon combustion. We will highlight that the understanding of the surface defects is key for the understanding of carbon combustion in general. Huge scientific efforts have been made to describe the fundamental reaction steps, and knowledge has progressed significantly over the past century. In this review we attempt to give a general overview of the aspects of the fundamental reaction steps which have been well understood and also what might still today pose a challenge for the scientific community.

## 4 Thermodynamics of Carbon Gas Reactions

Given the choice explained above to focus this review only on the reaction of pure carbon with oxygen, the two main chemical reactions are the following[^122^, ^171^]

(1) $$2\;C\left(\mathrm{graphite}\right)+O{}_{2}\left(g\right)=2\;\mathrm{CO}\left(g\right)$$

(2) $$C\left(\mathrm{graphite}\right)+O{}_{2}\left(g\right)=\mathrm{CO}{}_{2}\left(g\right)$$

Two secondary and / or concurrent reactions must be considered as well:

(3) $$2\;\mathrm{CO}\left(g\right)+O{}_{2}\left(g\right)=2\;\mathrm{CO}{}_{2}\left(g\right)$$

(4) $$C\left(\mathrm{graphite}\right)+\mathrm{CO}{}_{2}\left(g\right)=2\;\mathrm{CO}\left(g\right)$$

Here, the chemical compounds are considered in their stable phase under standard conditions, i. e. gas for O_2_, CO and CO_2_ and graphite for carbon.

From common experience corroborated by data in the literature,[^172^, ^173^] the standard enthalpies ▵H^0^ of reactions (1) to (3) are negative, which means that these reactions are exothermic.

The calculation of the standard Gibbs energies ▵G^0^ of reactions (1) to (3) for 1 mole of O_2_ indicates that these energies are strongly negative over a very wide temperature range from ambient temperature to more than 3500 K, which implies that there is no limitation of thermodynamic origin to these reactions.

**Figure 7:**
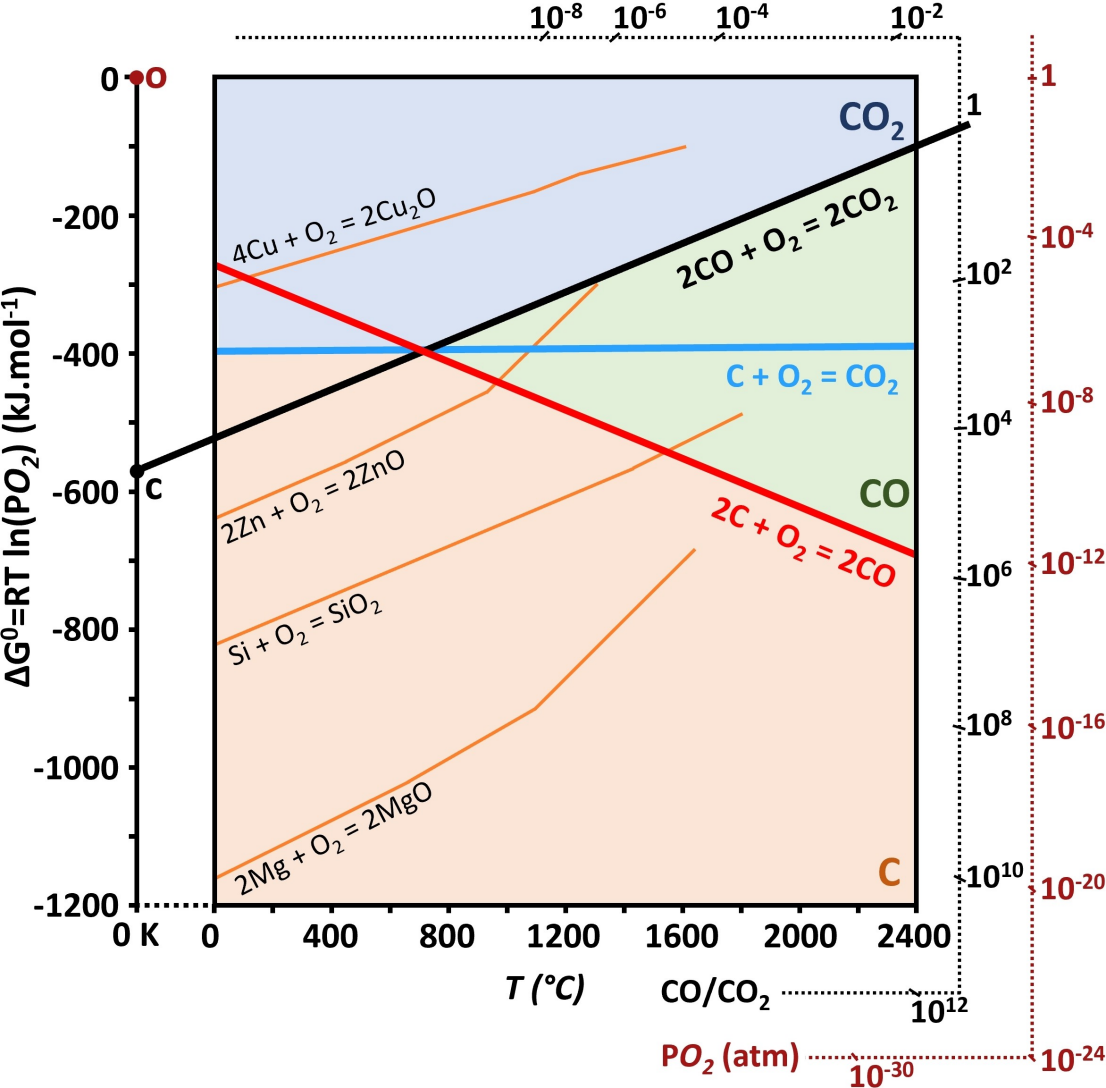
Ellingham diagram (standard Gibbs free energy versus the temperature, for the oxidation/reduction) of few species (P=1 atm). The predominance domains of C (orange), CO (green) and CO_2_ (blue) are represented. The red legend reports the equilibrium oxygen pressure. It also corresponds to the minimal oxygen pressure needed to oxidize an element at a given temperature (dry corrosion pressure). To read it, a line in between the diagram origin point (O in red) and the point of interest (element / temperature) should be reported on the scale. Taking the black C point on the ΔG^0^ as origin, the same method should be used to read the CO/CO_2_ ratio from the corresponding scale. Adapted with permission from Ref. [175]. Copyright 2022, Wiley‐VCH.

As these reactions are of very great interest in particular in the field of metallurgy (carbon reduction of metal oxides), it is usual to find the associated thermodynamic quantities ▵G^0^ represented in the form of an Ellingham diagram (Figure 7).[^174^, ^175^]

The blue line in Figure 7, relating to reaction (2), is almost horizontal, and thus the entropy term is low since the number of moles of gas is unchanged. On the contrary, the red line of reaction (1), due to the formation of one mole of gas, is strongly decreasing. These two curves cross at a temperature around 700 °C. For this temperature, at equilibrium, the partial pressure of oxygen p_O2_ is approximately 10^−22^ atm and the ratio of partial pressures of CO and CO_2_ (p_CO_ / p_CO2_) is equal to 1 (black line). Above this temperature the ratio increases and CO presents an increased stability compared to CO_2_.

A complementary way of presenting this dependency for combustion is by combining reactions (1) and (2) in the following form:

(5) $$\left(1+f\right)\;C+O{}_{2}=\left(1\;\text{-}\;f\right)\;\mathrm{CO}{}_{2}+2\;f\;\mathrm{CO}$$

In this equation (5), the factor f linked to the reaction parameters (temperature, total pressure, composition of the system) is dimensionless and can vary between 0 and 1. When f tends towards 1, the reaction (1) becomes predominant and thus formation of CO is maximized. Conversely, if f tends towards 0, the production of CO_2_ is preponderant and the global reaction is dominated by reaction (2).

Considering the remarks on the Ellingham diagram, an increase in temperature induces an increase in the value of f and therefore in the p_CO_ / p_CO2_ ratio. There is a long scientific history of discourse related to this question and the quest to identify if both CO/CO_2_ are primary products involving well‐known scientists such as I. Langmuir, J. R. Arthur, H. Eyring, P. L. Walker and others. We will return later to the importance attributed by certain authors to this p_CO_/p_CO2_ ratio on boundary layer diffusion as well as fundamental reaction steps (chapter 5, chapter 6).

Temperatures above 700 °C are of importance as here begins the region where the Gibbs energy of reaction (4), also known as the Boudouard reaction,^[176]^ becomes negative. This means that for an experimental device (oven, thermogravimetric analysis (TGA), etc.) in which a gas mixture rich in CO is cooled below 700 °C, the activity of carbon can become greater than 1 and that CO disproportionation can occur spontaneously, leading to carbon nucleation. The Boudouard reaction is thus used for the synthesis of soot (nucleation in homogeneous phase with high supersaturation in CO)^[177]^ but also of graphite crystals or carbon nanotubes (heterogeneous and / or catalyzed nucleation)^[178]^ and is currently exploited heavily for the production of novel, nanosized carbon allotropes.[^179^, ^180^]

To conclude, the thermodynamic analysis of the carbon‐oxygen system indicates that carbon oxidation is possible whatever the temperature. At low temperature, oxidation will essentially lead to the formation of CO_2_ while at high temperature CO will be preponderant. Oxidation is spontaneous from the thermodynamic point of view, the observation of the very slow reactivity (e. g. graphite crystals) demonstrates that kinetic aspects dominate the reactivity. This is connected to the question of rate determining step and activation energy and will be discussed in more detail in the following chapters.

## 5 Carbon Combustion, a Solid/Gas Surface Reaction

Combustion is a solid gas type reaction, and thus transport phenomena are critical for the understanding of this reaction like all other solid‐gas reactions. When solid particles are exposed to a gas, typically a loss in pressure is observed, which is an issue for heterogenous catalysis for instance. This is related with the filling of the micro/nanostructure of the solid in general, and needs to be considered in all type of fixed or fluidized bed reactors.^[181]^ Additionally, a boundary layer can be found in solid‐gas type reaction scenarios, situated between the surface of the particle and the bulk gas phase,^[182]^ in which a gradient of mass and/or temperature appears.^[183]^ This layer is always present whereas its thickness depends on the gas flow rate^[182]^ and the convection flow.

**Figure 8:**
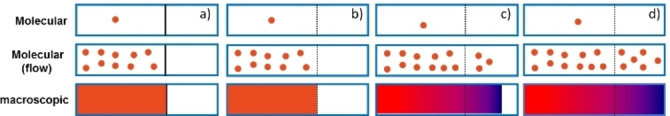
Schematic progress from a molecular, over a directed molecular flow to a macroscopic understanding of a bulk gas phase and a boundary layer. a) impenetrable barrier b) penetrable, with a very large chemical resistance c) the barrier is penetrable, out of equilibrium, some random passing of the barrier depending on the relative energy d) equilibrium situation, where the situation can be described by Fick's laws and the flow experiences a chemical resistance by the barrier.

Noteworthy, it does not depend on the oxygen concentration in the bulk gas phase.^[183]^ Consequently, the higher the flowrate, the thinner the boundary layer.^[182]^ Within the boundary layer, the mass transport of oxygen and convection is distinctively different from the bulk gas phase, leading to the term diffusion. In principle, two different approaches have been used to understand diffusion, a macroscopic picture and a molecular perspective, which has been conceptionally depicted in Figure 8.

**Figure 9:**
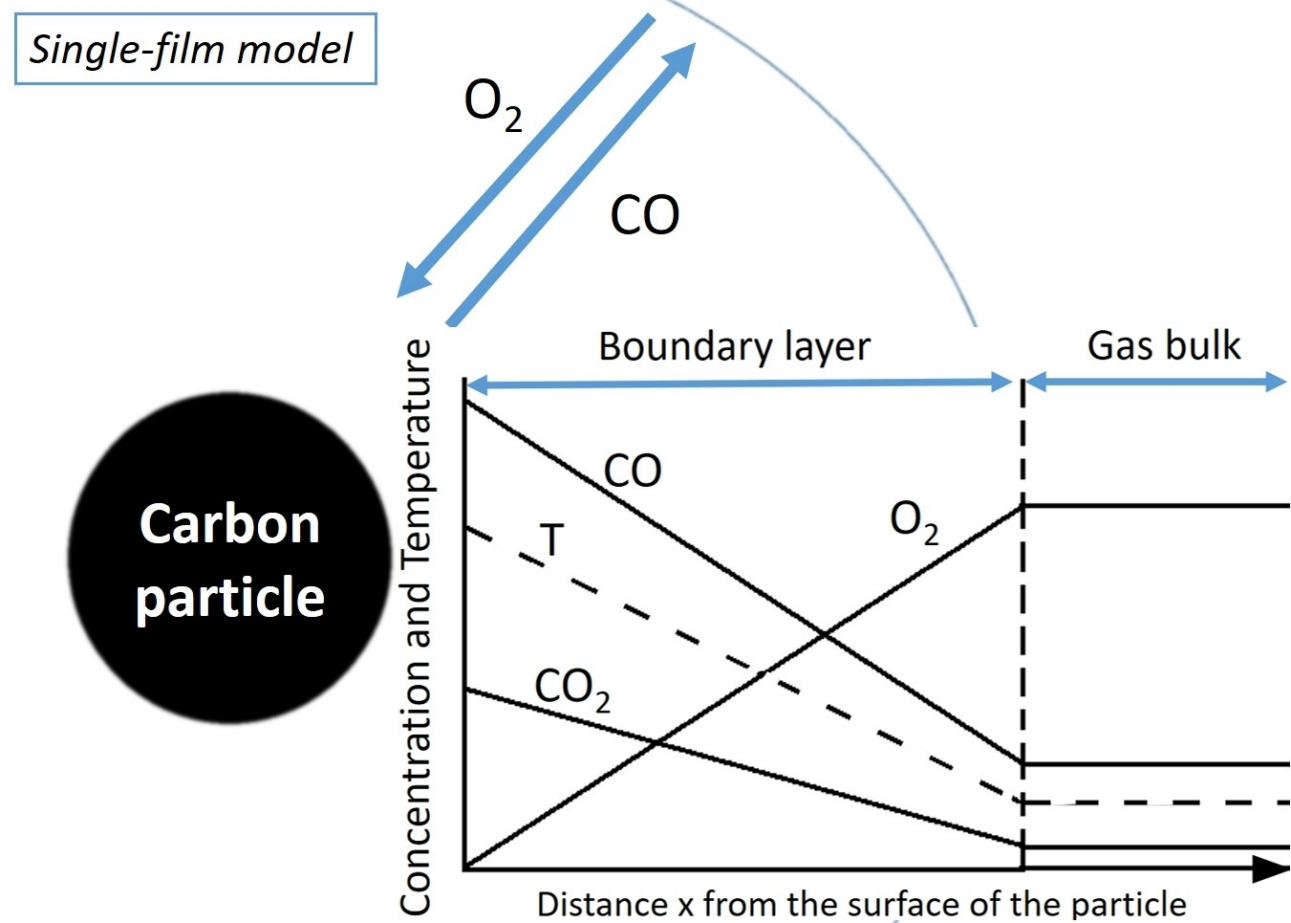
Single film model containing a stationary boundary layer and a bulk gas flow. In the flow the concentration of the educt and the product is constant, whereas in the boundary layer a concentration gradient for both the educt and the products can be found. This is dependent on the flow and the temperature and constitutes a physical limitation (see chapter 6 for a complementary discussion).

The microscopic point of view in a more general sense has been pioneered by De Gennes and others. It describes diffusion based on a random walk model, where the structure of the surface generates potential walls (energy gaps), that alter and confine the movement of each molecule.[^184^, ^185^, ^186^] Consequently, a detailed knowledge of the surface is crucial for a deep understanding at the microscopic point of view. The macroscopic picture is based on the Fick's law which itself is based on the general idea that the flow progresses from a high concentration area to an area of low concentration being exposed to a resistance. Consequently, a concentration gradient is formed which is proportional to the chemical resistance the gas is experiencing while traveling through the boundary layer. This resistance is directly related to the temperature (as the height of the potential wall).^[63]^ For the relative concentration of reactant in the boundary layer, the surface reaction plays a central role. Diffusion limitation is more likely at higher temperatures in all types of solid‐gas reactions. The thickness of the boundary layers depends on the balance between the viscosity and the flow rate, both for laminar as well as turbulent flows and thus influences both the mass and heat transport.^[181]^ In steady‐state, the reaction consumes the oxygen at the surface, which locally provokes a drop of oxygen concentration. The formation of boundary layers depends strongly on the reaction conditions, flow rate, oxygen concentration, temperature, etc., but also on the type of carbon materials for example porosity, meso/microstructure, particle size and last but not least on reactors shape and geometries.[^187^, ^188^] Carbon combustion generates both CO and CO_2_ as primary products as discussed above. A simple concentration boundary layer is represented in Figure 9 and the interested reader might be referred to more involved models taking opposite diffusion of oxygen and products into account (double boundary layers model).[^176^, ^181^, ^183^]

When the dioxygen molecule is close enough to the carbon atoms, it may adsorb on the surface. This chemisorption can occur only on an external layer of carbon, and preferentially on specific locations called the active sites (defects, edges, etc, cf chapter 6). Dioxygen molecules attached to the active sites are more or less mobile onto the surface, and can undergo chemical reactions forming adsorbed products. The products subsequently desorb leaving an active site free behind and the combustion reaches steady‐state. It shall be noted that the desorbed gaseous products may also experience diffusion limitation.^[122]^ Diffusion limitation can be either internal or external depending whether it occurs in the nano and meso‐pores or on the outer surface of the larger aggregates. In the case of internal diffusion limitation, a reduced concentration of oxygen in the particle is observed leading to a situation where not all active sites are used.^[181]^ In contrast, in external diffusion limitation, the effective oxygen concentration on the outer surface is reduced. The last case is especially important if the particles are exhibiting only a limited porosity (e. g. graphite crystals) and the chemical reaction is very fast (e. g. high temperature). One frequent model to understand the equilibrium between adsorption/desorption is the Langmuir‐Hinshelwood model. It basically describes the competition between the adsorption of oxygen on the surface and the desorption of products (CO, CO_2_) on the surface‐active sites. One of the easiest formulations of the Langmuir‐Hinshelwood model is the following,^[69]^ with R_global_ the global reaction rate, K_d_ and K_a_ the rate constants for the desorption and adsorption process respectively (Arrhenius form), and P_O2,s_ the oxygen partial pressure at the carbon surface:

(6) $$Rglobal=\frac{k_{d}k_{a}P_{O_{2,s}}}{k_{d}+k_{a}P_{O_{2,s}}}$$

Two limit cases should be considered, where either the adsorption or the desorption is the limiting step. If the adsorption is the limiting step (K_a_≪K_d_) the reaction rate is of first order for the oxygen, and can be described as follows R_global_=K_a_ P_O2,s_. If on the contrary the desorption of product is limiting the kinetics (K_d_≪K_a_, i. e., saturation of active sites by the products) the rate appears as a zero‐order reaction which can be expressed as follows R_global_=K_d_.[^71^, ^187^] Diffusion limitation effects can be diminished in some cases by a careful choice of the reaction parameters. The most frequently used methods consist in either decreasing the relative speed of the chemical reaction by decreasing its temperature or by increasing speed of motion of the oxygen molecules through the diffusive layer by changing the flow rate of the bulk gas. The latter case is strongly connected to the Fick law. Interestingly, carbon combustion data compatible with a kinetic limitation are obtained in the vast majority at relatively low temperatures (below 600 °C).[^78^, ^179^] Activation energies can only be obtained in the kinetic regime and if the reaction is either diffusion limited or adsorption/desorption limited, only apparent activation energies and apparent order of reaction can be obtained. If experimentally a change in apparent reaction rate is observed while varying the flow rate, an external diffusion limitation is very likely. On the contrary, if diffusion limitation is internal, no significant effect is to be expected by a total flow rate increase.^[190]^ In the literature, it is often possible to find dimensionless numbers, such as the effectiveness factor or the Thiele modulus, to distinguish between transport phenomenon or the chemical reactions and help to find the rate limiting step.^[181]^ The Thiele modulus originates from studies of heterogeneous catalytic reactions with a solid porous catalyst and directly compares the intrinsic reaction rate to the effective diffusion speed.^[191]^ If the Thiele modulus tends towards 0, a kinetic regime is present. In case it tends towards infinity, combustion is limited by diffusion.^[71]^ Depending on the geometrical model, describing the micro structure discussed above, different formulation of the Thiele modulus need to be applied.[^124^, ^179^] Mass transfer can be limited by the geometry of the porous network of the particles,^[192]^ and it strongly depends on the tortuosity of the porous network.^[193]^ Here, as in the case mentioned above the concentration of oxygen decreases gradually towards the center of the particle.^[192]^ The occurrence of internal diffusion is complex and several models are commonly used like Darcy's law or Knudsen number.[^65^, ^192^] The distribution and presence of different pore sizes like micro‐pores (less than 2 nm), meso‐pores (between 2 nm and 50 nm), and macro‐pores (larger than 50 nm) in a material is one central aspect. Another one is the adsorption/desorption equilibrium of the gaseous species within these pores, since in carbon combustion 3 different gaseous species are involved (CO, CO_2_ and O_2_). It is also noteworthy that the means of diffusion change when the length scale of the system is comparable to the mean free path of gaseous species involved. This is known as Knudsen diffusion, and plays a large role in the combustion of porous carbon materials, such as carbon blacks. It should also be kept in mind that in very porous carbon materials, the Fick laws might not be valid anymore due to the enhanced particle interactions. Carbon particles can be modelled as many spheres in contact with each other. Heat transfer distinguishes between two cases, *i)* inter and *ii)* intra particle. Interparticle heat transfer is related to the amount of contacts and typically only a small proportion contributes whereas the majority behaves as high thermally resistant regions.^[182]^ Intraparticle heat transfer occurs through lattice phonon vibrations within those carbon spheres.^[195]^ Consequently, the structure of the carbon material is of crucial importance. This propagation of heat is directly related to the length of the mean free path of phonon‐phonon interaction of the carbon lattice.^[195]^ If the length of this mean free path increases, the thermal diffusivity increases, thus favoring heat transfer.^[195]^ Likewise, thermal conductivity of coal increases with an increase in temperature during oxidation.^[195]^ Thus, internal heat conduction within the particle has more importance than oxygen diffusion if the particles are large and are of minor importance in very porous carbon materials.^[192]^ The internal temperature of carbon particles during combustion may be thus very different from the reactor temperature and should simultaneously be determined during kinetic experiments.^[194]^

## 6 Fundamental Reaction Steps from a Chemical Point of View

There is a large number of articles in the literature and a complete overview of the different approaches, models, and fundamental reaction steps in various fields (e. g. coal gasification or carbon fiber stabilization) exceeds the scope of this review. In this section we attempt to provide a brief overview over the most up to date concepts and try to collect the combined knowledge of various fields into a unifying concept that may be applied for different purposes such as the oxidation of HOPG up to the oxidative unzipping of carbon nanotubes as proposed by Radovic et al. in their recent works.[^14^, ^196^, ^197^, ^198^]

### 6.1 Reaction steps

**Figure 10:**
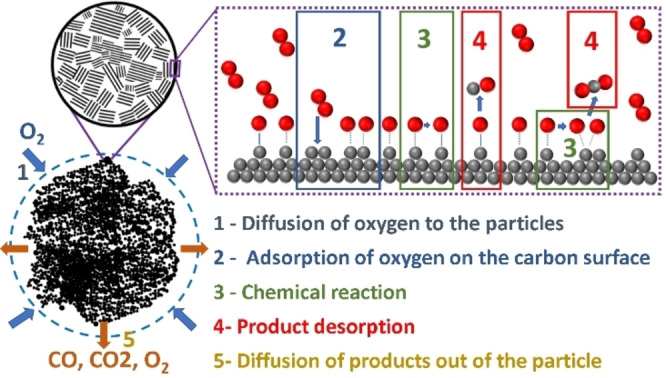
Scheme of the carbon combustion reaction (1) diffusion of oxygen to the carbon surface through the boundary layer (including pore diffusion, see chapter above). (2) Adsorption of oxygen atoms onto the surface (see chapter above) (3) Splitting of dioxygen and successive reaction steps forming the primary product CO or CO_2_ (discussed in this chapter) (4) Desorption of primary products from the surface (see chapter above) (5) Diffusion through the boundary layer of CO/CO_2_ (see chapter above).

Carbon oxidation typically follows successive crucial reaction steps (Figure 10).[^116^, ^124^, ^126^, ^198^, ^199^]

- Diffusion of oxygen to the carbon surface through the boundary layer (including pore diffusion, see chapter 5).
- Adsorption of oxygen atoms onto the surface (see chapter 5)
- Splitting of dioxygen and successive reaction steps forming the primary product CO or CO_2_ (discussed in this chapter)
- Desorption of primary products from the surface (see chapter 5)
- Diffusion through the boundary layer of CO/CO_2_ (see chapter 5)

**Figure 11:**
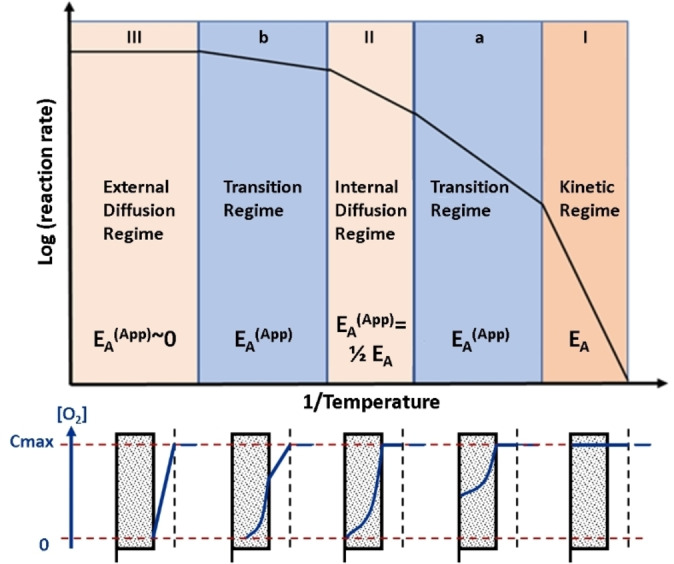
Kinetic and diffusive regimes by Walker.^[122]^ In the lower part the oxygen concentration in the bulk gas phase and the boundary layer (external and internal) is depicted. Only in the kinetic regime, the concentration of oxygen is identical in all regions and only there activation energies can be obtained. In all other regions only apparent activation energies can be measured as here physical limitation such as diffusion are rate limiting. Adapted with permission from Ref. [122]. Copyright 2022, Wiley‐VCH.

The dioxygen molecule first has to reach the carbon surface by diffusion through the concentration boundary layer (blue dotted circle on Figure 10). Depending on the temperature range, the reactor geometry and most importantly the effective concentration of oxygen on the reactive sites, the rate limiting steps are either chemical or diffusion processes. In 1959, Walker et al. presented a model where they postulated 3 defined regimes, one kinetic and two diffusion controlled regimes (Figure 11).^[122]^

The diffusion‐controlled regimes are split into internal (pore diffusion) and internal/external diffusion control (film diffusion), depending if the relative concentration of oxygen reaches zero in either the pore structure (Regime II) or the boundary layer (Regime III) as discussed in Chapter 5 and shown in Figure 11 bottom part. Diffusion limitation (Regime III) is occurring readily at high temperatures at which the surface reaction rates are faster than the diffusion of oxygen through the boundary layer (further details can be found in the seminal article of Walker).^[122]^ When internal diffusion limits the combustion and the concentration of oxygen in the internal structure reaches effectively 0, an apparent activation energy of exactly one half of the kinetic value is obtained (Regime II).^[65]^ The kinetic and the two diffusion regimes are bridged by transition regimes, where a complex behavior is to be expected. This can be readily understood by a reduced for example diminished concentration of oxygen between the boundary layer and the concentration in the bulk flow. In fact, there is a widespread confusion in literature about the values of activation energies and some authors even concluded that it is not possible to obtain kinetic parameters for the carbon combustion.^[201]^ Having the concept of apparent activation energy in mind, (see chapter 5) it is by no means surprising that over the last century, values for activation energies from close to zero to about 450 kJ.mol^−1^ have been obtained for the combustion of carbon materials deemed to be very similar such as coals. This observed apparent erratic behavior may also be fueled by the habit of classifying carbon materials, for example chars, coals and cokes without carefully analyzing the structural properties, as well as the porosity for example nano and mesostructures. Semi global intrinsic models are among the most developed techniques to explain various phenomena for example order of reaction.^[202]^ The interpretation of experimentally obtained apparent activation energies and the assignment to the correct regime (either kinetic or diffusion) is neither obvious nor straightforward and requires a broad dataset over a range of temperatures. We have recently shown that the majority of reported data for various carbon materials are neither in the internal diffusion regime nor in the kinetic regime but in a transition regime which complicates both the interpretation and the comparison of apparent activation energies.^[9]^ Thus, it is of crucial importance to obtain datasets over a broad temperature and oxygen partial pressure range and to keep simultaneously in mind that the obtained data may very likely lay in a transition regime. A similar observation can be made for the order of reaction where typically values between 0 and 1 have been reported. However, in recent years a change of the order of reaction was observed by a few authors including us, which can not be explained by the commonly used models.[^9^, ^79^, ^202^, ^203^] The observed order of reaction is also influenced by diffusion and thus by no means a fixation or assumption of 0 or 1 ought to be done *a priori*.

### 6.2 From reaction steps towards a mechanistic understanding: the role of active sites

The combustion reaction is occurring on defined positions and not randomly on the basal plane as shown by various authors over time. The most impressive examples are given by STM and SEM studies on freshly cleaved HOPG, see Figure 5. Here the formation of defined rings after exposure to oxygen at elevated temperatures can be observed.[^14^, ^17^, ^23^, ^203^, ^204^] These observations confirm the hypothesis that the fundamental reaction cascade is starting on active sites, also known as active surface area (ASA), a minor content of the total surface area in highly crystalline samples.[^129^, ^208^] Active sites are those on which chemisorption of the oxygen molecules occurs. ASA can be edges,^[208]^ missing lattice carbon atoms, diene type defects, heteroatoms,^[209]^ and Stones‐Wall defects^[210]^ amongst others.

In the 1950’s,[^141^, ^212^] for the first time ^18^O isotope labelling has been used to identify primary reaction products.^[124]^ From various experiments over time, it was concluded that both CO and CO_2_ are primary reaction products, which can be understood by the thermodynamics (see Chapter 4).^[212]^ From in‐depth IR, temperature desorption, and X‐ray Photoelectron spectroscopy (XPS) measurements it was concluded that oxygen adsorbs on carbon surfaces, then dissociates and form oxygen complexes on the surface.[^205^, ^213^, ^214^, ^215^, ^216^] These surface oxygen complexes are found to be mobile on the carbon surface,[^217^, ^218^, ^219^] and are crucial for the carbon combustion reaction.^[132]^ Interestingly, it was found that a part of those surface complexes cannot be thermally desorbed even at temperatures above 900 °C.^[143]^ This is an important point for the elucidation of the correct mechanism because consequently not all chemisorbed oxygen is able to take part in the carbon combustion reaction. Consequently, it should be kept in mind that oxygen complexes may be ultimately very stable and do not lead to carbon‐carbon bond cleavage of the sigma bonds in a hexagonal carbon lattice by themselves. During reaction, the reactive sites are those that participate directly in chemisorption of oxygen and formation of products and consequently the term reactive surface area (RSA) is commonly used. The reactivity of the discussed different types of defects or ASA may be ultimately very different initially, and attempts have been made to classify these defects in more detail. Moreover, RSA may also depend on temperature and relative burn‐off ratio.[^148^, ^220^, ^221^] The most important fundamental question, still disputed today, relates to the exact chemical nature of the reactive sites and if different ASA leads to different RSA. Importantly, it has to be kept in mind that carbon combustion reaches steady state if the temperature and other reaction parameters are kept constant. Mechanistic discussion thus needs to propose reactive sites that are present and are formed throughout the course of carbon combustion. Moreover, proposed mechanism must not only account for the formation of the reactive sites, but should be equally applicable to all forms of *sp* ^2^‐hybridized carbon materials: graphite, nanotubes, pyrolytic carbons, filamentous carbons, chars, cokes, activated carbons, and soot.

There are various examples published of how to determine the amount of ASA or RSA in a given samples using methods such as temperature programmed desorption (TPD), TGA, O_2_ or I_2_ adsorption or XPS.[^71^, ^222^, ^223^, ^224^] However, each of these methods has its advantages and shortcomings and the less ordered the carbon material, the less comparable the results are. Thus, a more general procedure may be desired that serves as a standard to determine ASA/RSA for various types of carbon material and to allow their comparison. Nevertheless, by normalizing the rate of combustion by the amount of ASA the rate constant of different graphite materials basically merges, a very strong argument that the reaction occurs almost exclusively on defects.[^129^, ^206^, ^225^] More modern models for example oxygen transfer model by Windes and co‐workers take into account the ASA and the dynamics of the oxygen complex formation.[^71^, ^77^]

### 6.3 From reactive sites towards fundamental reaction steps

**Figure 12:**
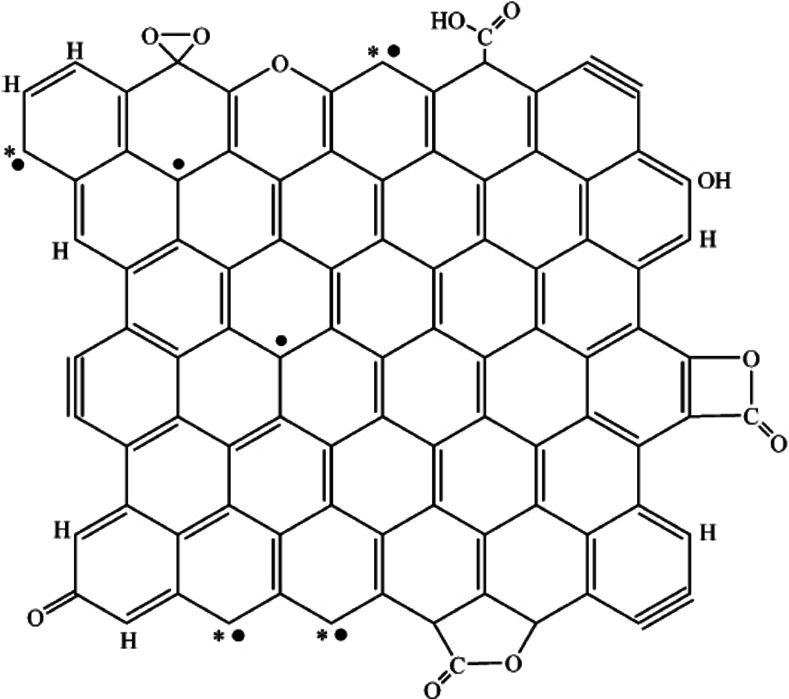
Schematic representation of the main chemical features in a graphene sheet, with its typical surface functionalities, including free edge sites. The pairing of σ (*) and π (•) electrons at the zigzag sites and the presence of triple bonds at the armchair sites is indicated. The abundance of aromatic sextets and the degree of π electron delocalization depends on size, shape, and connectivity of the graphenes, as well as on their edge termination. Adapted with permission from Ref. [209]. Copyright 2022, Wiley‐VCH.

**Figure 13:**
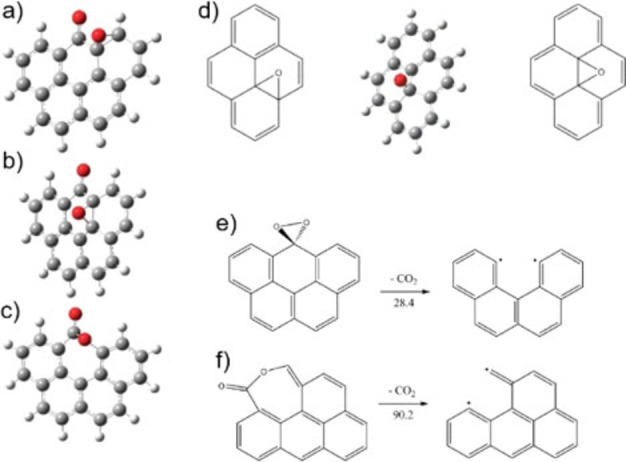
a),b),c) Optimized geometries of carbon clusters representing the indirect path to CO_2_ formation involving different transition states d) mechanism for O‐hopping (surface diffusion) on the graphene basal of the oxygen surface complex e,f) direct versus indirect mechanism and thermochemistry of CO_2_ desorption. The grey spheres represent the carbon atoms, red spheres the oxygens and the white, hydrogens. The ▵E values shown are in kcal/mol. The lactone structure is favored. Adapted with permission from Ref. [227]. Copyright 2022, Wiley‐VCH.

Radovic et al. published several articles over the last 2 decades revealing both by calculations and experimentally mechanistic details on carbon combustion and trying to combine the consequences arising from various studies of surface complexes and RSA.[^208^, ^210^, ^218^, ^226^, ^227^] The understanding of what is a defect and how does this defect contribute to chemical reactivity is a central aspect not only for carbon combustion but also in the ongoing quest of understanding reactivity of novel nanocarbon allotropes.[^49^, ^196^, ^198^, ^208^, ^228^, ^229^, ^230^] Carbon edges in *sp* ^2^ carbon lattices can be either armchair or zig‐zag, the latter being the more reactive one in general.[^71^, ^231^] Stable hydrogen‐saturated graphene edges may also be present. Due to the extended solid‐state carbon lattice structure of graphene (or other allotropes), defects such as carbine like or carbene like unadulterated radicals formed by H removal from graphene edges might be reasonably stable (Figure 12).^[208]^ Isolated carbene‐type zigzag carbon atom and a carbyne‐type armchair pair of carbon atoms are proposed as chemically reactive sites in carbon materials by Radovic (see also Figure 12).^[227]^ This hypothesis may account also for a remarkable variety of physicochemical observations for example unique amphoteric behavior of carbon materials, positive thermoelectric power, and ferromagnetism.^[227]^ The most important mechanistic arguments may be summarised as follows: *i)* oxygen complex may serves as a reservoir of reactive oxygen species*, ii)* the reaction is initiated at defects, such as zig‐zag edges under the formation of a quinone or semiquinone structure, *iii)* a cascade of rearrangement reactions is occurring, involving the oxygen complexes nearby, that leads to the breaking of one sigma C−C basal plane carbon bond under the formation of a lactone *iv)* steady state regime where the formation of reactive sites is constant *by releasing of the product from the carbon surface* generating a constant concentration of reactive surface sites *v)* reaction of basal plane carbons are very unlikely at lower temperatures Lactone formation during the course of oxidation of carbon materials have been observed experimentally from early on by different methods, for example IR‐spectroscopy or XPS (see Figure 13).[^212^, ^232^] Lactone formation is thus understood to be an important reaction step in the fundamental reaction cascade occurring during carbon combustion. The lactone structure supports two arguments: firstly, the formation of CO_2_ does not involve the concomitant breaking of two sigma carbon lattice bonds but just one and secondly the carbon oxygen bonds are established in an earlier step. The quinone and semiquinone structures were studied by means of calculations, to ascertain whether they may provide a direct pathway to CO. It was found that albeit possible, the energy demand for this reaction is high.[^233^, ^234^] However, these arguments may explain why CO is formed as a product only above a certain temperature and typically only in a low oxygen regime. Predominantly CO_2_ is formed at lower temperatures and carbon combustion progresses for prolonged time, even if the oxygen supply is stopped.^[14]^ This can only occur if an oxygen reservoir is present on the surface feeding the surface reaction. Thus, the surface complex (see Figure 13) is crucial for the carbon combustion. Radovic et al. have argued that the reaction step resembles the benzene oxide/oxepin tautomerism,^[217]^ which has been established for benzene oxide and higher aromatic hydrocarbons showing that an equilibrium can be found in each case and is dependent on the molecular structure of the respective aromatic compound.^[235]^ All these arguments and experiments provide ample evidence that the oxygen surface complex and its mobility is a crucial component for carbon combustion reaction (see also Figure 13). The surface complex is thus connected directly to the formation of the lactone species and is involved through rearrangement reactions. The release of product molecules (carbon dioxide) leaves unpaired electrons behind, which are by themselves very reactive sites. As discussed those are presumably either carbene‐type zigzag carbon atoms or carbyne‐type armchair‐atoms. This formation is directly related to kinetics of the carbon combustion in case ample reactant is present. In case reactant concentration is insufficient, deactivation may occur due to rearrangement reactions. To summarise, at a given temperature, a quasi‐equilibrium can be reached, where for every CO_2_ release a certain amount of “new” reactive surface sites is generated, explaining the constant reaction rate under constant reaction conditions (e. g. temperature, partial pressure, flow profiles amongst others). The mechanistic understanding of the carbon combustion reaction has come a long way and a remarkably detailed picture has emerged. It has long been known that the Boudouard equilibrium favours CO over CO_2_ at high temperatures.^[236]^ One open question is if the proposed mechanism is also valid at higher temperatures for example diffusive regime and if a link can be potentially established to the related field of oxygen plasma etching of carbon forms, where CO is the predominant product.^[237]^ It has been observed by STM measurements on HOPG that the typical pit growth mechanism rapidly changes at around 900 °C where a drastic increase in number of pits has been observed.^[238]^ These experiments are important to describe the fundamental reaction steps occurring on the surface at higher temperatures, and more studies in the regime III are desired in order to solve these questions in the future.

## 7 Kinetic Measurements

While the thermodynamics is well understood, the kinetics of carbon material oxidation is still under study. This may be obvious when considering the structural variety of carbon materials and the consequence arising from boundary layer diffusion. In fact, for each carbon material, a quite unique kinetic profile may be obtained, depending on its structure and the experimental conditions. The most general kinetic equation to describe the reaction is:^[239]^

(7) $$\frac{d\alpha }{dt}=k\left(T\right)f\left(\alpha \right){PO}_{2}^{n}$$

with *α* the progress of reaction, k(T) a function of the temperature T, *f(α)* a function of *α*, *PO_2_ the* oxygen partial pressure and n the order of reaction for oxygen. This equation is only valid under atmospheric total pressure and a term accounting for the pressure should be added in case the reaction is performed in closed reaction vessels. The temperature dependency for the conversion rate is usually connected to the Arrhenius law, giving rise to equation 8. It introduces a pre‐exponential factor k_0_ and the Activation energy Ea. R is the universal gas constant.

(8) $$\frac{d\alpha }{dt}=k_{0}e^{-E_{a}/RT}f\left(\alpha \right){PO}_{2}^{n}$$

The aim of kinetic studies is then to determine the triplet of the factors E_a_, f(*α*) and n. Several formulations for the f(*α*) function can be found elsewhere and can either arise from empirical laws (e. g. power laws, giving rise to a fixed reaction order for the carbon) or derived from models of structural evolution of the material during its conversion (e. g. contracting or shrinking core models).[^69^, ^240^, ^241^] Often, several functions are compared and the one giving the best fit is kept. E_a_ (n) can then be obtained by studying the effect of temperature and/or oxygen partial pressure variations. This is achieved by its linearization or integration of equation 8.[^239^, ^242^]

**Figure 14:**
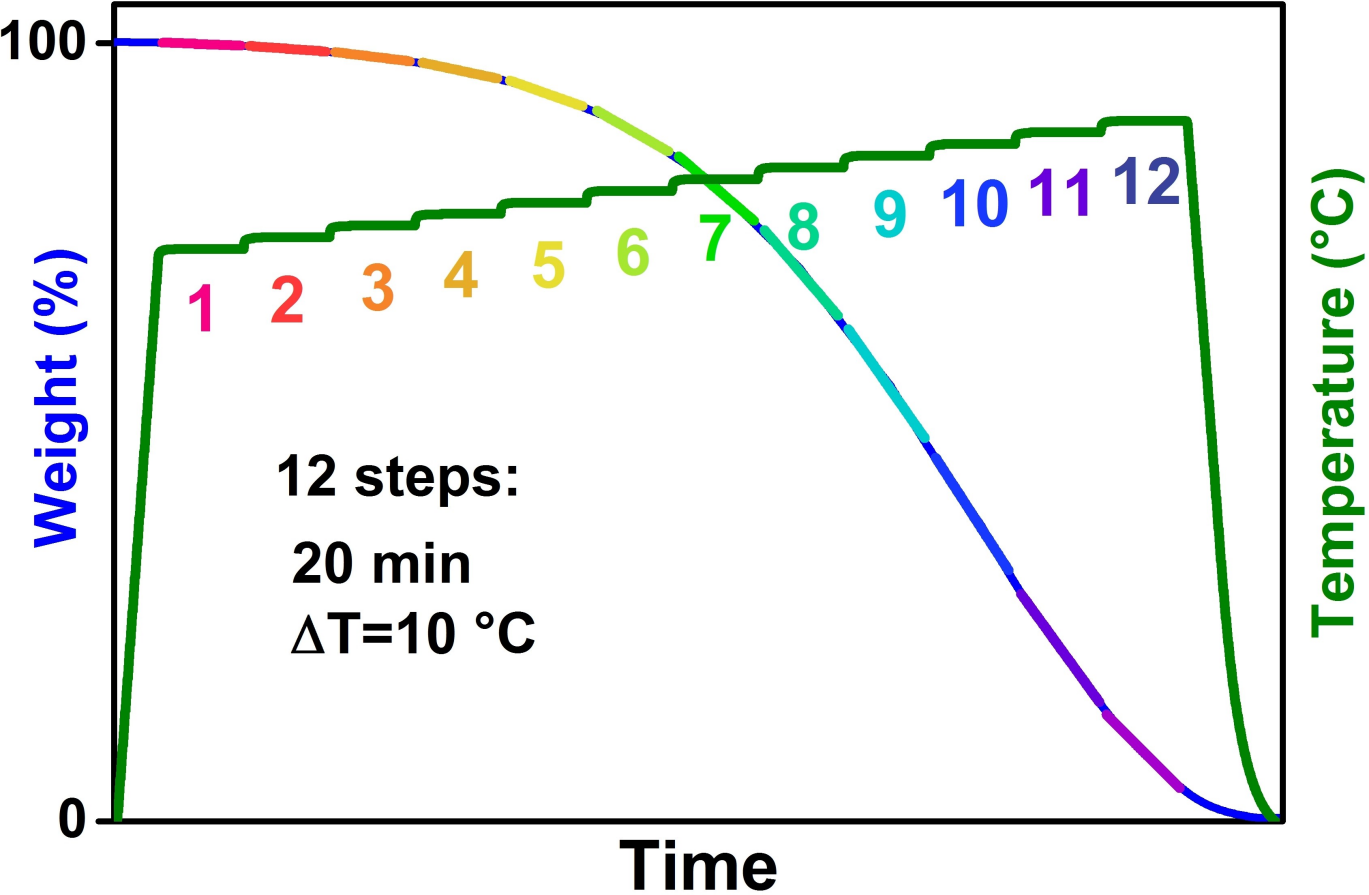
SIS program developed for the analysis of different carbon (nano) materials, allowing a kinetic analysis with one TGA experiment. Adapted with permission from Ref. [9]. Copyright 2022, Wiley‐VCH.

To carry out kinetic studies, the reaction is induced by a temperature programmed oxidation (TPO) while *α* is followed versus time. The most popular TPO are by far, the isothermal experiments and the ones applying a constant heating rates, but variation are known for example stepwise isothermal[^243^, ^244^] or successive isothermal steps.[^9^, ^245^] Stepwise isothermal, also called quasi‐isothermal or controlled transformation rate thermal analysis (CRTA) among others, is a dynamic technique in which the mass loss controls the temperature of the machine, instead of having a temperature profile imposed before the measurement. Such a method is particularly well adapted to determine the temperature of a phenomena (thermal decomposition, reaction temperature, etc.) but some variation have been proposed for kinetic studies purpose, for example the forced stepwise isothermal analysis (FSIA). It is in principle possible to get an E_a_ value by measuring TPO with a constant heating rate. However, only one measurement is not sufficient for a trustworthy value and the international confederation for thermal analysis and calorimetry (ICTAC) insists on the importance to perform multiple measurements to get reliable values.^[239]^ We recently proposed a new TPO, the successive isothermal steps (SIS).^[9]^ It consists in the concatenation of several finite time isothermal steps, close in temperature (see Figure 14). While the philosophy of this method is fairly close to the one of the FSIA, the SIS method can be readily applied in the simplest TGA machine. Typically, in SIS a succession of minimum 12 isothermal steps allows to construct an Arrhenius plot from one measurement only. The reaction conversion on each step can be analyzed as an independent measurement, giving the same precision as 12 individual isothermal measurements.

In order to monitor the progression of the carbon combustion, popular observables are the weight loss of the material (thermogravimetric methods), the geometrical change of the material (macroscopic shrinking, widening of microscopic pits, etc.) and the more or less direct gaseous products detection of CO/CO_2_. Combination of those methods have been employed as well, for example gas detection on the out exhaust of a thermogravimetric analysis.[^99^, ^187^, ^242^, ^246^]

As previously mentioned, due to the heterogeneous nature of the reaction, the true kinetic values can only be determined if the chemical surface reaction is the limiting step, i. e., the reaction progresses in the kinetic regime (zone I).^[122]^ This implies that in studies involving wide T and pO_2_ ranges, a change of the limiting step can occur, including diffusional limitations. However, the most crucial aspect is to carefully check if the combustion is truly in the kinetic regime, which is only possible by a large and detailed dataset. Thus, it is inevitable to measure and analyze also those data, which subsequently are in a transition regime and also in diffusion limitation regime.

## 8 Conclusion

Optimizing carbon combustion was never a purely chemists’ task but involved scholars, smiths and in later times, engineers. The approach towards optimizing carbon combustion has always been driven by the need of optimisation rather than detailed understanding. Albeit we are day by day moving further and further away of relying on carbon combustion for energy and heat production, this reaction still poses fundamental questions. This is due to the complexity and the interplay of different limitations such as diffusion, heat, mass transfer, and the number of active sites amongst others. From various studies over the years, it becomes clear that carbon combustion is occurring primarily on active sites, and not on the basal plane. The relative concentration of oxygen available on those active sites determines if the reaction is kinetically or diffusion controlled. It now becomes apparent that mostly diffusion influenced kinetic parameters have been obtained for the majority of carbon‐based materials (graphite, carbon black, chars, etc.). It is of fundamental importance in these cases to speak of apparent activation energy and apparent order of reaction. The authors of this review believe that more carefully conducted surface analysis as well as coupled measurements, especially in situ, should provide better understanding of the fundamentals of this challenging reaction. Content and nature of active sites (e. g. edge dislocations) should be described in a more detailed picture to understand their role in carbon combustion. Finally, all those insights may be readily used to understand the functionalization of carbon nanomaterials in greater detail, since concepts such as RSA and ASA are relevant to functionalization as well.

## 9 Perspectives

Looking back over centuries of research on carbon combustion, this reaction has literally fueled the development of mankind and truly astonishing developments have been made. This maybe best highlighted by the role of carbon materials for propulsion purposes that allows our current way of life, with all its so practical amenities. However, it has drastic consequences on our planet from the continuous emission of CO_2_ in the atmosphere and the resulting climate change. From a scientific point of view, the carbon combustion reaction is an endeavor facing similar highs and lows. From a basic aspect, remarkable progress in the understanding of structure, properties and chemical reactivity has been made, but still after centuries of research a unifying concept bringing all pieces together is missing. This is caused by the huge number of possible carbon structures which have fundamental consequences on diffusion, and resulting reactivity, amongst others. This aspect is especially important in respect to the high temperature region, not yet fully tackled (fundamental reactions at temperatures >1000 °C). Structural understanding might only be gained if attention is paid to the nano and micro‐structure of the carbon materials, which connects this topic to the ongoing activity in the field of 2D nanomaterials for example graphene. Working both in nanomaterial research and carbon combustion, the authors feel that both fields tend to overlook structural understanding in the search of potential applications, which may lead to culs‐de‐sac with a myriad of apparently contradictory studies, each on a unique material. An in‐depth structural analysis for each study is desirable, that is potentially rigorized and homogenized, in order to forge a comprehensive picture including defects, structure, porosity, as well as reactivity and may lead one day to a predictable, unifying concept that may be applied to the majority of carbon materials.

## Conflict of interest

The authors declare no conflict of interest.

10

## Biographical Information

*Emmanuel Picheau received in 2017 both an engineering degree from the Chemistry school of Bordeaux (ENSCBP) and a M.Sc. degree in Material Science from Bordeaux University. He then joined the Nanotube and Graphene team at CRPP and obtained his Ph.D. under Alain Pénicaud's supervision in 2020. His work focused on flattened carbon nanotubes and carbon combustion. In 2022 he joined Renzhi Ma's group at NIMS (Tsukuba ‐ Japan), under a postdoctoral JSPS fellowship (Standard)*.

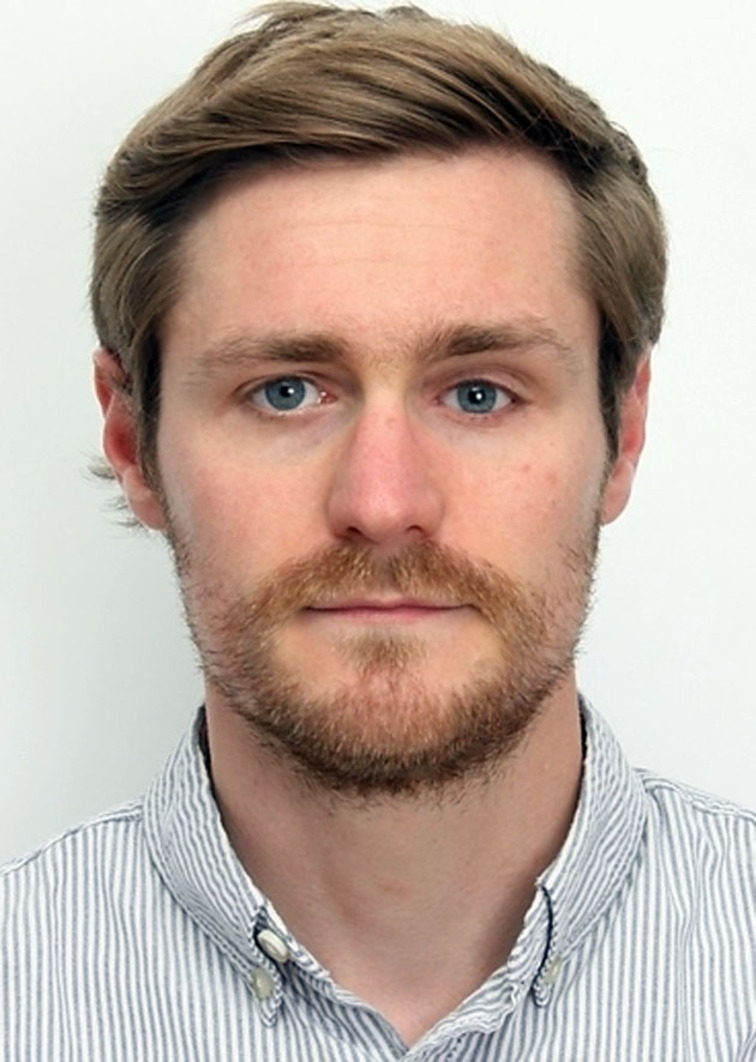

## Biographical Information

*Sara Amar obtained an engineering degree from the Institut Mines Telecom Lille Douai and has a brief work experience in the aeronautic industry. She received a Masters degree at the University of Bordeaux and is currently a Ph.D. student at the University of Bordeaux working on Zeolite‐Templated Carbon for energy storage applications under the supervision of Alain Pénicaud and Alain Derré*.

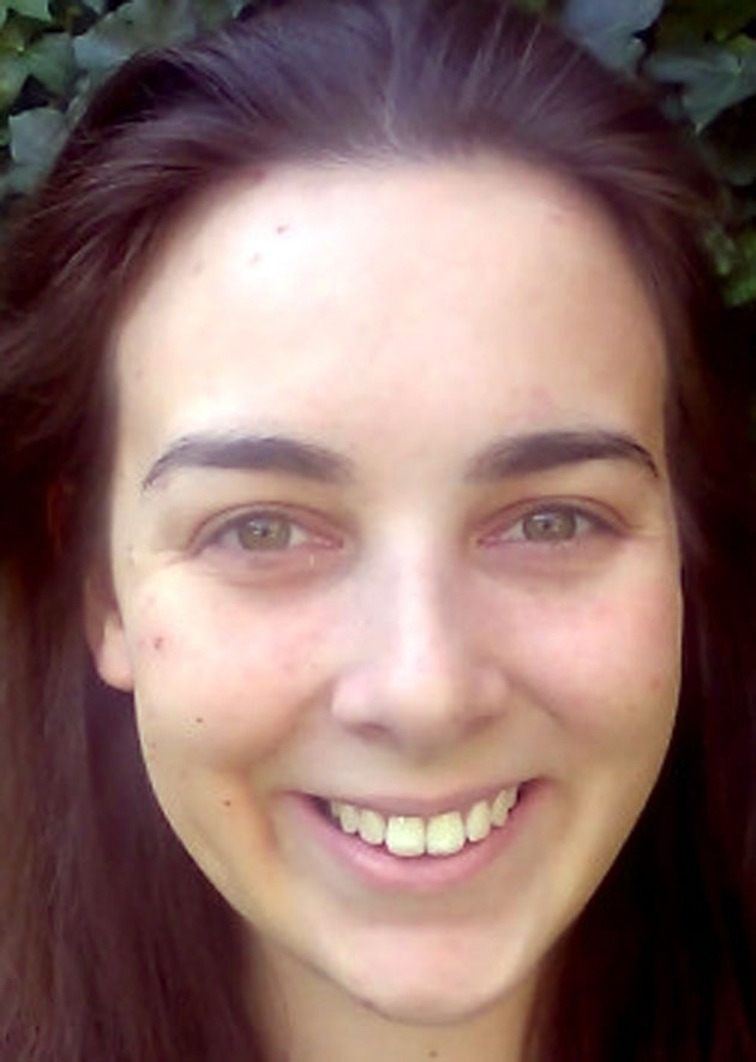

## Biographical Information

*Alain Derré is a CNRS researcher. After an engineering degree in technical ceramics (ENSCI‐Limoges‐1983) and a Ph.D. (University of Perpignan‐1988), he worked in Bordeaux in the field of refractory ceramic coatings as well as on various materials based on carbon, in particular nano‐composites with a polymer matrix and nanotube and / or graphene reinforcement*.

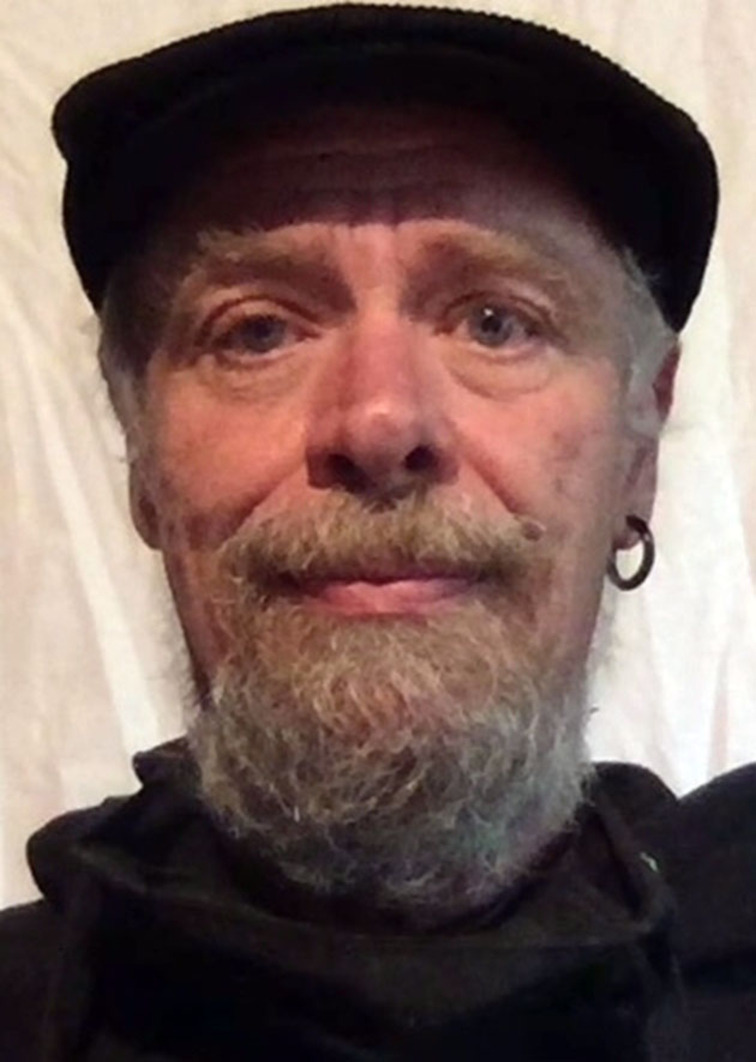

## Biographical Information

*Alain Pénicaud is a senior researcher at CNRS, France. After a Ph.D. from the University of Rennes (1988), and two years at the University of Southern California, he has worked, first in Mexico then in Bordeaux, using redox chemistry to crystallize soluble fullerenes or dissolve insoluble novel carbon nanoforms. Besides scientific papers on carbon science and patents, he has authored a popularization book on the history of crystallography and is co‐founder of a start‐up company, Carbon Waters, commercializing aqueous graphene formulations and coatings*.

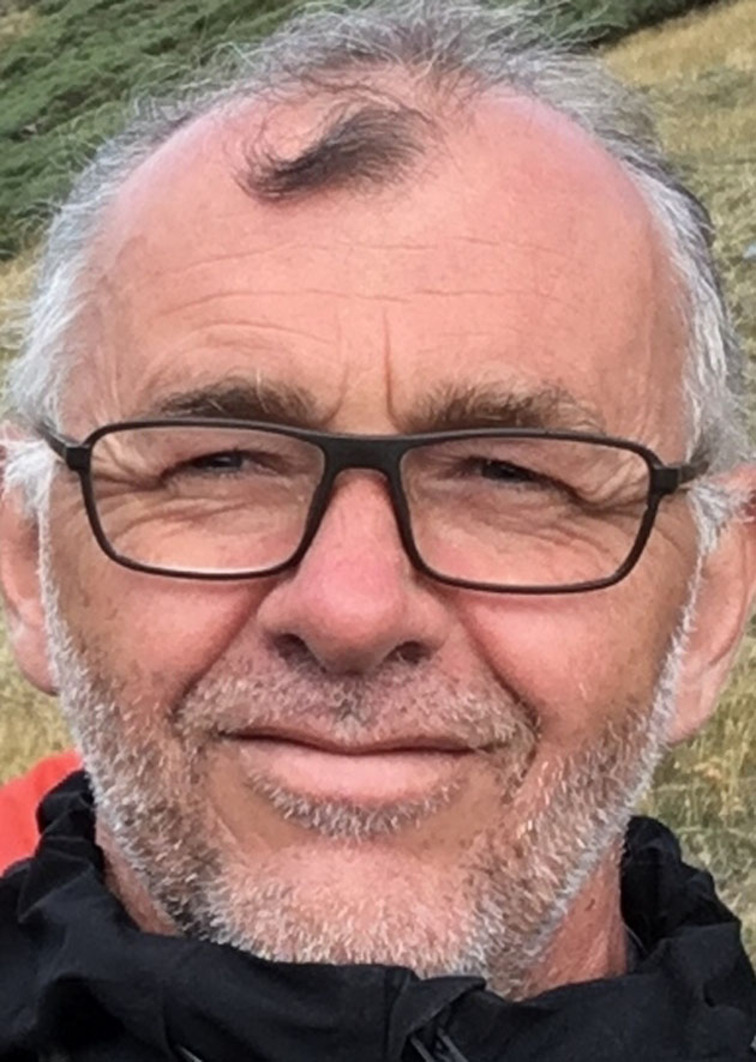

## Biographical Information

*Ferdinand Hof graduated from the Friedrich‐Alexander‐Universität Erlangen‐Nürnberg (FAU), Germany in 2010. He obtained his doctorate under the supervision of Prof. Dr. Andreas Hirsch (FAU) in 2015. He joined Alain Pénicaud group in 2015 as Post‐doc. He is currently independent researcher having obtained an individual research fellowship in 2017 (DFG) and the prestigious CNRS Momentum 2018. His work is focused on chemistry on carbon nanomaterials, synthesis of carbon nanoparticle composites, intercalation chemistry and carbon combustion*.

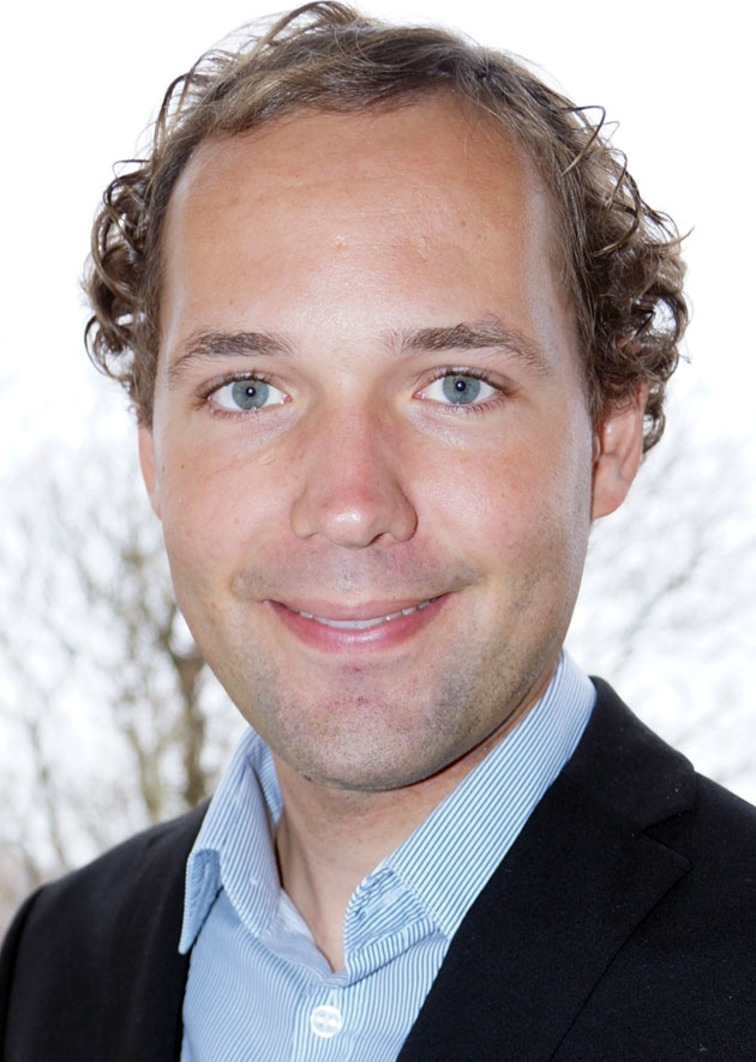

## Data Availability

Data sharing is not applicable to this article as no new data were created or analyzed in this study.
